# Palatine Tonsil Stenting of the Airway as Determined by Drug-Induced Sleep Endoscopy

**DOI:** 10.1155/2018/2614143

**Published:** 2018-12-10

**Authors:** Habib G. Zalzal, Steven Coutras

**Affiliations:** Department of Otolaryngology-Head and Neck Surgery, West Virginia University School of Medicine, Morgantown, WV, USA

## Abstract

**Objective:**

To demonstrate lateral pharyngeal wall collapse and increased apnea-hypopnea index in a child posttonsillectomy.

**Background:**

Some children have worsening of their sleep symptoms after tonsillectomy for obstructive sleep apnea. This case report demonstrates an open airway on drug-induced sleep endoscopy (DISE) in a child with tonsillar hypertrophy followed by more pronounced airway obstruction related to lateral pharyngeal wall collapse after tonsillectomy.

**Case Presentation:**

A 7-year-old boy presented with obstructive sleep apnea and underwent workup with DISE. Following adenotonsillectomy and subsequent lingual tonsillectomy with epiglottopexy, the patient's sleep apnea symptoms and polysomnogram results worsened. Subsequent DISE showed a more narrowed oropharyngeal airway space as compared to his preoperative DISE.

**Discussion:**

Palatine tonsillar tissue may splint open the airway and prevent airway obstruction in a subset of pediatric patients. Further clinical studies are necessary to determine which children experience this phenomenon. Clinical examination using DISE can be useful in making clinical decisions prior to tonsillectomy.

## 1. Introduction

Drug-induced sleep endoscopy (DISE) enables exploration of a patient's airway and is used particularly in patients with obstructive sleep apnea (OSA). First described in 1991, the pediatric procedure involves a flexible endoscope inserted through the nose of a sedated child [1, 2]. The endoscope is positioned at different upper airway levels to detect mechanisms and sites of obstruction [1, 2]. At present, DISE is restricted to children without hypertrophic tonsils and to those with persistent OSA following tonsillectomy and adenoidectomy (T&A) [2].

OSA affects up to 5.7% of all children and is widely recognized as a cause of significant morbidity [2–4]. Polysomnography is the most comprehensive method for diagnosis and evaluation of OSA severity [2, 4]. Adenotonsillar hypertrophy is an undisputed major contributor towards OSA in otherwise healthy children, such that the American Academy of Pediatrics recommends T&A as first-line therapy [2–4]. However, previous meta-analyses have noted 18%–33.7% of children who do not experience resolution of sleep apnea after this surgery, thus questioning the common attitude to systematically perform T&A in affected children [5, 6].

At our institution, we noted a few of our pediatric patients had worsened apnea-hypopnea index (AHI) on polysomnogram following T&A (Table 1). For one such patient described in this case report, we theorize a phenomenon that allows for hypertrophied tonsils to be nonobstructive, as the tonsils could instead splint or stent the airway open. This lends to the controversial belief that there may be a positive benefit for leaving tonsils in place.

## 2. Case Presentation

A 7-year-old boy presented to the otolaryngology sleep medicine clinic after being referred by his neurologist for OSA (Video 1). His past medical history was significant for a traumatic brain injury suffered after a dog attack during infancy, resulting in neurologic developmental delay, posttraumatic stress disorder, and attention deficit hyperactivity disorder. He underwent a polysomnogram prior to evaluation, which showed mild OSA with an AHI of 1.7, rapid eye movement (REM) AHI of 6.3, and a low oxygen saturation of 92%. On clinical examination, his tonsils were noted to be a +3 size (75% of oropharyngeal airway) on the Brodsky scale, but there was clinical concern that there may be additional sites of obstruction. He underwent a DISE with T&A one month later, and on postoperative follow-up, his mother did not believe his snoring had improved.

A few months later, based on initial DISE findings of a retroflexed epiglottis with lingual tonsil hypertrophy, he underwent an epiglottopexy with lingual tonsillectomy. A postoperative sleep study performed three months after that surgery (and five months after T&A) was concerning for worsening OSA (AHI 4.5; REM AHI 12.1; low O_2_ saturation 94%). He underwent a second postoperative DISE four months after that polysomnogram, which showed complete collapse of the base of the tongue against the posterior pharynx. When compared to his preoperative DISE prior to T&A, it became apparent that the tonsils were likely not obstructing at the time, but splinting the airway open. The patient was scheduled for a posterior midline glossectomy but was lost to follow-up.

## 3. Discussion

With the advent of DISE, the complexity of pediatric OSA has recognized that adenotonsillar hypertrophy is not the sole cause of sleep apnea [3]. Tonsil size itself has been shown to not be a predictor of OSA severity, as even hypertrophied tonsils do not result in worse sleep apnea for pediatric patients [7]. A 2009 meta-analysis by Friedman et al. showed that treatment success of T&A was only 66.3%. Although one out of every three children failed to have complete resolution of sleep apnea following T&A, the surgery still reduced AHI significantly for the majority of patients and thus was recommended as first-line therapy [6].

Additionally, children with small tonsils may not experience the same benefit of T&A compared to those with larger tonsils based on the Brodsky standardized system [8, 9]. A 2016 study by Miller et al. established the Chan–Parikh system of classifying tonsillar obstruction via DISE. Their study concluded that a Brodsky score of +1 tonsils did not consistently demonstrate lateral pharyngeal wall collapse based on their new DISE-based scoring system [9].

Much is known about small tonsils not causing oropharyngeal obstruction, but little has been written on hypertrophied tonsils doing the same. Hypertrophied tonsils have been known to cause velopharyngeal insufficiency in certain patients due to posterior placement of the upper poles of the tonsils into the oropharyngeal and nasopharyngeal airway [10–12]. However, to our knowledge, there are no recorded reports in the literature of enlarged tonsils splinting the airway open to allow for an improved airway during sleep. It can be surmised that if tonsils are large enough to create enough space within the velopharynx, then perhaps it can do the same for the oropharynx along the base of the tongue. In addition to the case report described above, several other patients in our clinic were found to have similar instances of this tonsillar physiology (Video 2). It is the author's expectation that our video imaging of this stenting phenomenon may explain one possible mechanism as to why some patients with large tonsils do not improve following tonsillectomy.

## 4. Conclusion

While splinting of the tonsils to open the airway may not be a common pathology, patient characteristics should always be addressed before performing a T&A. If there is concern that the tonsils are not the obvious source of obstruction on physical examination, a preoperative DISE may assess whether a tonsillectomy is the correct surgery for this patient. While there is no identifying characteristics at this time for who may have this tonsil-splinting phenomenon, our hope is that our case presentation and video imaging sheds light as to why some patients may not improve after tonsillectomy for pediatric OSA.

## Figures and Tables

**Table 1:** Characteristics of pediatric patients with worsened obstructive sleep apnea after tonsillectomy.

Patient (age/sex)	Comorbidities	Pre-DISE AHI	Procedures between polysomnogram	Recent AHI	Tonsil size (Brodsky scale)
7M^†^	ADHD, traumatic brain injury	1.7	Tonsillectomy and adenoidectomy; epiglottopexy; lingual tonsillectomy	4.5	3
7M	ADHD, fragile X	5.2	Tonsillectomy and adenoidectomy	9.4	3
7F	ADHD, Down's syndrome	4.9	Tonsillectomy and adenoidectomy; turbinate reduction	8.1	2

DISE, drug-induced sleep endoscopy; AHI, apnea-hypopnea index; ADHD, attention deficit hyperactivity disorder. ^†^This individual was the first patient noted to have had tonsillar splinting after review of sleep endoscopy posttonsillectomy.

## Supplementary Materials

Supplementary Materials Video 1: this video begins with tonsillar-splinting phenomenon in the case report of the patient. Preoperatively, the tonsils are stenting the oropharyngeal airway and remain in a static nonobstructing position throughout respiration. The epiglottis represents main source of obstruction. The latter half of the video shows postoperative airway after tonsillectomy and epiglottopexy. There is now collapse of the tongue base only months after initial surgery when the previous tonsillar positioning would prevent such obstruction by splinting the airway open. Video 2: Patient #1 shows static, splinting tonsils throughout respiration with obstruction secondary to retroflexed epiglottis. Patient #2 shows splinting tonsils preventing further collapse of the posterior base of the tongue against the epiglottis. Patient #3 shows enlarged tonsils preventing obstruction of the tongue against the retroflexed epiglottis. Subsequent endoscopy after lingual tonsillectomy and epiglottopexy shows continued tonsillar splinting, but obstruction now due to arytenoid collapse.
